# A Two-Degree-of-Freedom Knee Model Predicts Full Three-Dimensional Tibiofemoral and Patellofemoral Joint Motion During Functional Activity

**DOI:** 10.1007/s10439-022-03048-2

**Published:** 2022-09-09

**Authors:** Shanyuanye Guan, Hans A. Gray, Lucas T. Thomeer, Marcus G. Pandy

**Affiliations:** Department of Mechanical Engineering, University of Melbourne, Parkville, VIC 3010 Australia

**Keywords:** Knee-joint complex, Hinge joint, Kinematic coupling, Secondary motions

## Abstract

Six kinematic parameters are needed to fully describe three-dimensional (3D) bone motion at a joint. At the knee, the relative movements of the femur and tibia are often represented by a 1-degree-of-freedom (1-DOF) model with a single flexion–extension axis or a 2-DOF model comprising a flexion–extension axis and an internal–external rotation axis. The primary aim of this study was to determine the accuracy with which 1-DOF and 2-DOF models predict the 3D movements of the femur, tibia and patella during daily activities. Each model was created by fitting polynomial functions to 3D tibiofemoral (TF) and patellofemoral (PF) kinematic data recorded from 10 healthy individuals performing 6 functional activities. Model cross-validation analyses showed that the 2-DOF model predicted 3D knee kinematics more accurately than the 1-DOF model. At the TF joint, mean root-mean-square (RMS) errors across all activities and all participants were 3.4°|mm (deg or mm) for the 1-DOF model and 2.4°|mm for the 2-DOF model. At the PF joint, mean RMS errors were 4.0°|mm and 3.9°|mm for the 1-DOF and 2-DOF models, respectively. These results indicate that a 2-DOF model with two rotations as inputs may be used with confidence to predict the full 3D motion of the knee-joint complex.

**Supplementary Information:**

The online version contains supplementary material available at 10.1007/s10439-022-03048-2.

## Introduction

Six parameters are needed to fully describe the relative positions of the bones which meet at a joint. At the knee, the three-dimensional (3D) movements of the tibia relative to the femur are usually described by the tibiofemoral flexion angle, which represents the primary motion of the knee, together with 5 secondary motions comprising external rotation, abduction, anterior translation, lateral shift, and joint distraction. Similarly, the 3D movements of the patella relative to the femur are described using another set of 3 rotations and 3 translations. Thus, at most 12 kinematic parameters are needed to describe all relative movements of the femur, tibia and patella at the knee. However, some or all of these parameters may be related (or coupled) to each other due to the constraints imposed by the knee ligaments, capsular structures, and articular contact at the joint.^2,6,19,21^

Experiments on intact cadaver knees suggest that the relative movements of the femur and tibia may be described using two simultaneous rotations occurring about fixed axes.^11^ Tibiofemoral flexion–extension occurs about a mediolateral axis fixed in the femur and passing close to the centers of the femoral condyles, whereas internal–external rotation of the tibia occurs about a longitudinal axis fixed in the tibia and passing through the medial tibial plateau. These two rotations, tibiofemoral flexion and external tibial rotation, which define a two-degree-of-freedom (2-DOF) kinematic system, were found to adequately describe the 3D movements of the femur and tibia during weightbearing activity simulated *in vitro*.^3^ Wilson *et al.*^25,26^ challenged this proposition by showing that all five secondary motions of the tibia traced the same paths relative to the femur when cadaver knees were moved passively in unloaded flexion and extension, implying that the tibiofemoral joint has 1-DOF. Sancisi and Parenti-Castelli^21^ showed further that the 3D movements of the femur, tibia and patella may be reproduced during passive (unloaded) flexion using a 1-DOF spatial kinematic mechanism with the tibiofemoral flexion angle specified as an input.

More recent *in vivo* studies have found that some, but not all, of the secondary motions of the tibia are coupled to the tibiofemoral flexion angle during seated knee extension^5^ and level walking.^12,13^ In our previous work, mobile biplane X-ray imaging was used to measure all 12 tibiofemoral (TF) and patellofemoral (PF) kinematic parameters for a wide range of daily activities.^23^ We found that 7 of the 11 secondary motions of the bones—3 translations at the TF joint, 3 translations at the PF joint, and patellar flexion—were coupled to the TF flexion angle. Importantly, external tibial rotation was only weakly related to the TF flexion angle. These results support the view that a kinematic model with more than 1 DOF is needed to accurately describe 3D bone motion at the knee.^3,11^

The main aim of the present study was to determine the accuracy with which 1-DOF and 2-DOF knee models predict the full 3D movements of the femur, tibia and patella during dynamic activity. A 1-DOF model with the TF flexion angle as the only input and a 2-DOF model with TF flexion and external tibial rotation as inputs were constructed by fitting polynomial functions to all 12 TF and PF kinematic parameters measured for 6 functional activities: level walking, downhill walking, stair ascent, stair descent, and open-chain knee flexion and extension. Multivariate regression was performed to investigate how well each model described the kinematic behavior of the knee-joint complex across all 6 activities. A ‘leave-one-out’ cross-validation analysis was then conducted to evaluate the accuracy with which the 1-DOF and 2-DOF models predicted 3D TF and PF joint kinematics across all 6 activities. We hypothesized that a 2-DOF kinematic model with TF flexion and external tibial rotation as inputs would predict all remaining secondary motions of the tibia and patella more accurately than a 1-DOF kinematic model with only the TF flexion angle specified. A kinematic model that accurately predicts the 3D movements of the femur, tibia and patella may improve existing musculoskeletal models that use skin-marker measurements obtained from video motion capture to estimate muscle and joint loading at the knee.^4^

## Materials and Methods

### Participants

Ten healthy individuals (6 males and 4 females; age 29.8 ± 6.1 years; height 168.0 ± 9.9 cm; weight 68.3 ± 9.0 kg) with no knee pain and no history of lower-limb surgery gave informed consent to participate in this study. Ethics approval for the experimental procedures was obtained from the Human Research Ethics Committee at the University of Melbourne.

### Experimental data

**Figure 1:**
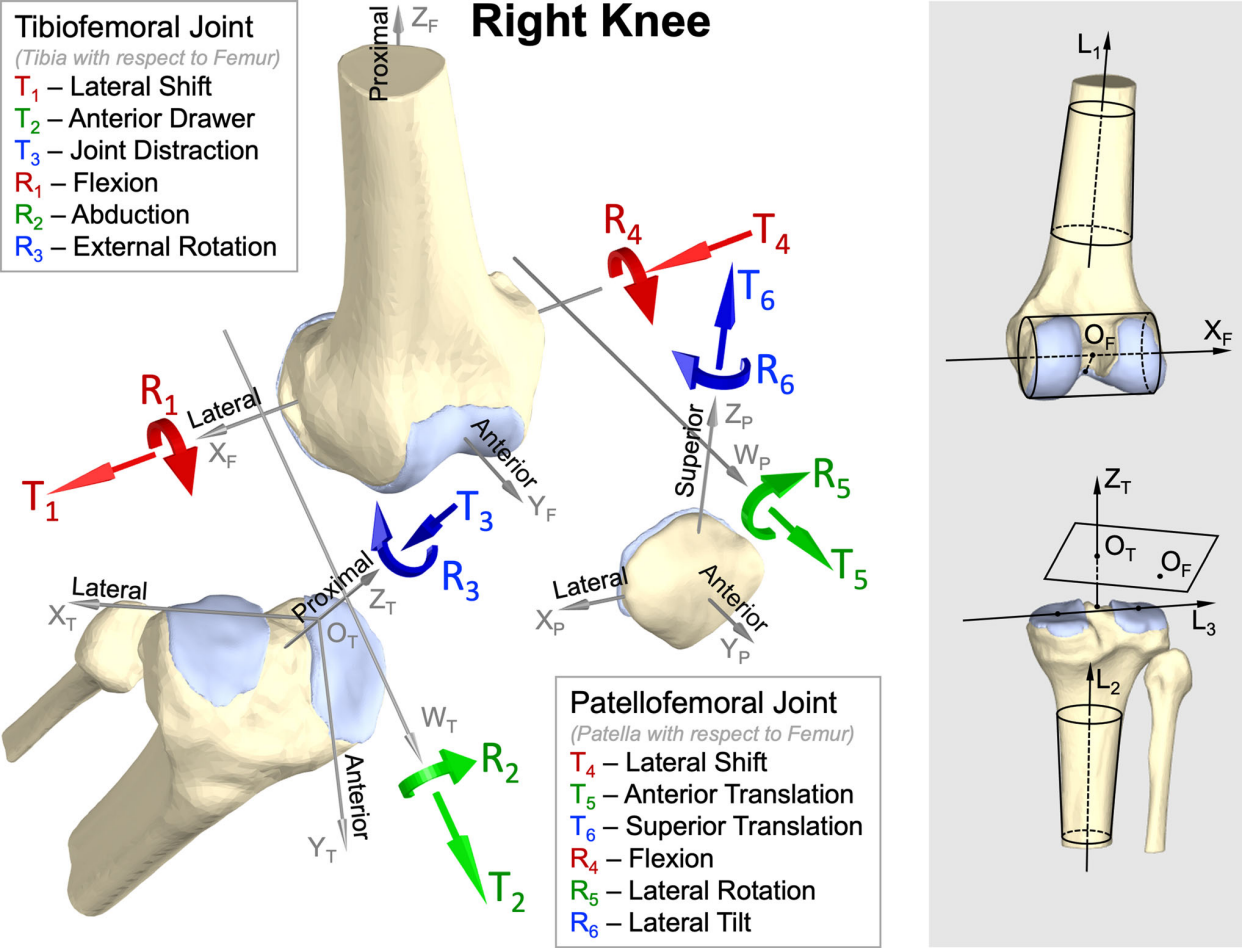
Twelve kinematic parameters were used to describe the complete three-dimensional motion of the bones at the tibiofemoral (TF) and patellofemoral (PF) joints of the right knee. Coordinate systems were assigned to the femur, tibia and patella using the convention adopted by Gray *et al*.^9^ The femoral coordinate system was constructed by fitting a cylinder to the posterior and distal portions of both femoral condyles (top-right panel). The *X*-axis (*X*_F_) of the femur was defined as the axis of the cylinder pointing to the right. A long axis (*L*_1_) pointing proximally was determined by fitting a cone to the femoral diaphysis. The *Y*-axis (*Y*_F_), which was mutually perpendicular to *L*_1_ and *X*_F_ and pointed anteriorly, was found by taking the cross-product of these two vectors, thus *L*_1_ × *X*_F_. The *Z*-axis (*Z*_F_) was mutually perpendicular to *X*_F_ and *Y*_F_ and pointed proximally; it was located by taking the cross-product *X*_F_ × *Y*_F_. The origin (*O*_F_) of the femur was located at the foot of the perpendicular from the intercondylar notch apex to *X*_F_. For the tibial coordinate system, a long axis (*L*_2_) was found by fitting a cone to the tibial diaphysis, and a vector (*L*_3_) pointing to the right was defined by a line joining the approximate center of each tibial plateau (bottom-right panel). The *Z*-axis (*Z*_T_) of the tibia was parallel to *L*_2_, pointed proximally, and passed through the midpoint between the two intercondylar eminences. The *Y*-axis (*Y*_T_) was mutually perpendicular to *Z*_T_ and *L*_3_ and pointed anteriorly; it was located by taking the cross-product *Z*_T_ × *L*_3_. The *X*-axis (*X*_T_) was mutually perpendicular to *Y*_T_ and *Z*_T_ and pointed to the right; it was located by taking the cross-product *Y*_T_ × *Z*_T_. The origin (*O*_T_) of the tibia was determined using the relative pose between the tibia and femur during a CT scan with the knee unloaded and fully extended. The origin (*O*_T_) was located at the intersection of *Z*_T_ and the plane that passed through *O*_F_ and was perpendicular to *Z*_T_. For the patellar coordinate system, the *Y*-axis (*Y*_P_) of the patella was defined as the principal axis of inertia and pointed anteriorly. The *X*-axis (*X*_P_) was mutually perpendicular to *Y*_P_ and the posterior patellar ridge and pointed to the right. The *Z*-axis (*Z*_P_) was mutually perpendicular to *X*_P_ and *Y*_P_ and pointed superiorly; it was located by taking the cross-product *X*_P_ × *Y*_P_. The origin (*O*_P_) of the patella was defined as the centroid of the patella. Six kinematic parameters at the TF joint were defined by two body-fixed axes *X*_F_, *Z*_T_ and one floating axis, *W*_T,_ which was mutually perpendicular to *X*_F_ and *Z*_T_. Similarly, six kinematic parameters at the PF joint were defined by two body-fixed axes *X*_F_, *Z*_P_ and one floating axis, *W*_P,_ which was mutually perpendicular to *X*_F_ and *Z*_P_. The positive direction defined for each kinematic parameter is indicated by a red, green or blue arrow.

6-DOF TF and 6-DOF PF joint kinematics for a range of activities of daily living were reported previously by Thomeer *et al.*,^23^ where details relating to data collection and processing are also given. Each participant performed open-chain knee flexion–extension (125.9 ± 21.0 deg/s), level walking (1.31 ± 0.14 m/s), downhill walking (0.84 ± 0.09 m/s), stair ascent (0.63 ± 0.09 m/s), and stair descent (0.62 ± 0.05 m/s). Open-chain knee flexion–extension was performed with the participant standing on the left leg and flexing the right knee to lift the right foot off the ground. Knee flexion and knee extension were treated as two separate activities even though data were collected for a single continuous cycle of flexion–extension. Downhill walking was performed on a wooden surface sloped at 10 degrees relative to a level laboratory floor, while stair ascent and stair descent were performed on a wooden staircase comprised of 17 cm high steps (see Fig. 1 in Thomeer *et al*.^23^ for details). Biplane X-ray images (1024 × 1024 pixels, 200 frames/s, 1/200 s exposure time) of the right knee were acquired using a Mobile Biplane X-ray (MoBiX) imaging system.^10^ The biplane X-ray images were then imported to custom software in MATLAB (MathWorks Inc., Natick, MA) to perform pose-estimation of the femur, tibia and patella and calculate TF and PF joint kinematics. Geometric models of each bone required for pose estimation were created from CT scans (0.35 × 0.35 × 0.50 mm) taken of the right knee. Coordinate systems were assigned to the femur, tibia and patella to describe joint kinematics in an anatomically meaningful way^9^ (Fig. 1). Maximum root-mean-square (RMS) errors associated with 3D kinematic measurements of the intact knee were reported previously to be 0.78 mm and 0.77° for translations and rotations of the TF joint^10^ and 0.37 mm and 1.46° for translations and rotations of the PF joint.^8^

TF and PF joint kinematics measured simultaneously for 60 motion trials (1 trial per activity and 6 activities for each of the 10 participants) were analyzed in the present study. Kinematic data for each trial were filtered using a fourth-order, low-pass, Butterworth filter with a cut-off frequency of 10 Hz and then resampled to 201 time points to create 201 observations. Each observation contained 12 kinematic parameters: lateral shift, anterior drawer, joint distraction, flexion, abduction, and external rotation at the TF joint; and lateral shift, anterior translation, superior translation, flexion, lateral rotation, and lateral tilt at the PF joint (Fig. 1). TF and PF translations were normalized by the ratio of femoral bicondylar width measured for each participant to the mean femoral bicondylar width calculated across all 10 participants (81.7 mm)

### Multivariate Regression

Multivariate regression was performed to determine how accurately 1-DOF and 2-DOF models describe the kinematic behavior of the knee-joint complex across all 6 activities. Second-order polynomial equations were used to fit the complete set of 3D kinematic data (i.e., 12 kinematic parameters for the TF and PF joints combined) obtained from biplane X-ray imaging (see Section S2 of the Supplementary Material, which provides a justification for the use of second-order polynomials). Each polynomial equation described the relationship between the input TF kinematic parameters (e.g., TF flexion angle) and one of the remaining kinematic parameters defining either TF or PF joint motion (e.g., patellar flexion). A 1-DOF model consisted of 11 second-order polynomial functions with the TF flexion angle defined as the input variable. Each polynomial described one of the 11 secondary kinematic parameters ($y$) as a function of the TF flexion angle and took the form:

1 $$y = c_{0} + {\text{c}}_{11} f + {\text{c}}_{21} f^{2}$$

where $f$ is the TF flexion angle, and $c_{0}$ and ${\text{c}}_{ij}$ are constant coefficients. Similarly, a 2-DOF model consisted of 10 second-order polynomials, with each polynomial defined as a function of two input variables: TF flexion and external tibial rotation. Here, each polynomial described one of the 10 remaining kinematic parameters ($y$) and took the form:

2 $$y = {\text{c}}_{0} + {\text{c}}_{11} f + {\text{c}}_{12} e + {\text{c}}_{21} f^{2} + {\text{c}}_{22} e^{2}$$

where $f$ is the TF flexion angle, $e$ is external tibial rotation, and $c_{0}$ and ${\text{c}}_{ij}$ are constant coefficients. The coefficients in each model were found using a least-squares method. Each polynomial in the 1-DOF and 2-DOF models was created by fitting 12,060 observations obtained by pooling data from all 60 motion trials (201 time points per trial and 6 trials for each of the 10 participants). For individual participants, 1-DOF and 2-DOF models were also created by fitting 1206 observations obtained by pooling data from all 6 activities for each participant (201 time points per trial and 6 trials for each participant).

To determine how accurately each model described the kinematic behavior of the knee-joint complex, a fitting residual for each polynomial was calculated for the TF joint and PF joint separately for the 1-DOF and 2-DOF models. RMS residuals for the TF joint (${\text{RMSR}}_{{{\text{TFJ}}}}$) were computed as follows:

3 $${\text{RMSR}}_{{{\text{TFJ}}}} = \sqrt {\frac{1}{{\left( {6 - n} \right)p}}\mathop \sum \limits_{i = 1}^{6 - n} \mathop \sum \limits_{j = 1}^{p} \left( {y_{ij} - y^{\prime}_{ij} } \right)^{2} }$$

where $y_{ij}$ and $y_{ij}^{^{\prime}}$ are, respectively, the fitted and measured values of each output TF kinematic parameter (indexed by $i$) for each observation (indexed by $j$); $p$ is the number of observations; and $n$ is the number of input TF kinematic parameters (i.e., the number of DOFs of the model, $n = 1 \;{\text{or}} \;2$). Similarly, RMS residuals for the PF joint (${\text{RMSR}}_{{{\text{PFJ}}}}$) were obtained as follows:

4 $${\text{RMSR}}_{{{\text{PFJ}}}} = \sqrt {\frac{1}{6p}\mathop \sum \limits_{i = 1}^{6} \mathop \sum \limits_{j = 1}^{p} \left( {y_{ij} - y^{\prime}_{ij} } \right)^{2} }$$

where $y_{ij}$ and $y_{ij}^{^{\prime}}$ are, respectively, the fitted and measured values of each PF kinematic parameter (indexed by $i$) for each observation (indexed by $j$); and $p$ is the number of observations. Rotations of 1° and translations of 1 mm were weighted equally at both the TF and PF joints, and the notation 1°|mm was used to indicate a residual value of 1° or 1 mm. The model that more accurately described the kinematic behavior of the knee-joint complex was the one associated with lower residuals at the TF and PF joints.

### Model Cross-Validation

A ‘leave-one-out’ cross-validation^1,18^ was conducted to evaluate the accuracy with which a 1-DOF model and a 2-DOF model predicted the 3D movements of the femur, tibia and patella across all activities. Second-order polynomials were again used to create 1-DOF and 2-DOF models by fitting the kinematic data for 54 trials obtained for 9 of the 10 participants (1 trial per activity and 6 activities for each of the 9 participants). Each model was then used to predict 3D TF and PF kinematics for the one remaining (left-out) participant. This procedure was performed 10 times by ‘leaving out’ a different participant’s data on each occasion. Model accuracy was quantified by calculating the errors between the predicted and measured values for each TF and PF output kinematic parameter.

## Results

### Multivariate Regression

**Figure 2:**
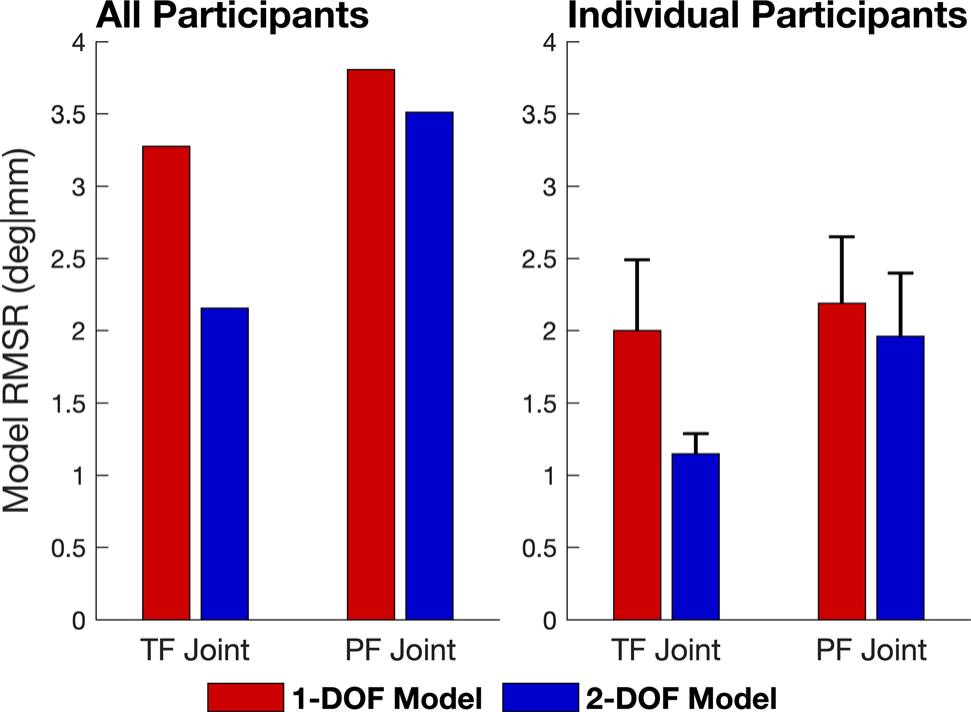
Root-mean-square residuals (RMSR) calculated for the 1-DOF and 2-DOF models at the tibiofemoral (TF) and patellofemoral (PF) joints. Only those residuals associated with the kinematic parameters predicted by each model were used to calculate the RMSR (i.e., kinematic parameters used as input variables were not included). Rotations of 1° and translations of 1 mm were equally weighted. The left panel shows the results obtained when each model was fitted using data from all 6 activities and all 10 participants (60 trials) pooled. The right panel shows the results obtained when each model was fitted using data from all 6 activities (6 trials) pooled from each participant. In the right panel, an RMSR was calculated for each participant, and the mean (height of the colored bar) and standard deviation (error bar) of the RMSRs across all participants were then found.

When data from all participants were pooled, RMS residuals for the 1-DOF model were 3.3°|mm at the TF joint and 3.8°|mm at the PF joint compared to 2.2°|mm at the TF joint and 3.5°|mm at the PF joint for the 2-DOF model (Fig. 2, left panel). Thus, increasing the number of DOFs from 1 to 2 reduced the residual by 1.1°|mm (33%) at the TF joint and by 0.3°|mm (8%) at the PF joint. A similar trend was observed when data from all 6 activities were pooled for each participant (Fig. 2, right panel). In this instance, increasing the number of DOFs from 1 to 2 reduced the residual by 0.9°|mm (45%) at the TF joint and by 0.2°|mm (9%) at the PF joint. Polynomial equations defining the 1-DOF and 2-DOF knee models are presented in Table 1.

**Table 1:** Polynomial functions defining the 1-DOF and 2-DOF models of the knee-joint complex comprising the tibiofemoral and patellofemoral joints.

		**1-DOF Model**			**2-DOF Model**				
	Equation	$y = c_{0} + c_{11} f + c_{21} f^{2}$			$y = c_{0} + c_{11} f + c_{12} e + c_{21} f^{2} + c_{22} e^{2}$				
	Input variables	$f$—tibiofemoral flexion angle (deg)			$f$—tibiofemoral flexion angle (deg) $e$—external tibial rotation (deg)				

	Kinematic parameter ($y$)	$c_{0}$	$c_{11}$	$c_{21}$	$c_{0}$	$c_{11}$	$c_{12}$	$c_{21}$	$c_{22}$
*Tibiofemoral Joint*									
Translations (mm)	Lateral shift	1.57	− 0.0488	1.37E−04	1.54	− 0.0585	− 0.029	2.00E−04	2.35E−03
	Anterior drawer	0.58	0.0390	4.74E−04	0.51	0.0014	− 0.155	7.32E−04	4.30E−03
	Joint distraction	− 1.74	0.0161	− 3.47E−04	− 1.76	0.0220	0.053	− 3.96E−04	2.61E−03
Rotations (deg)	Flexion		1			1			
	Abduction	− 4.79	− 0.0213	3.79E−04	− 4.87	− 0.0567	− 0.136	6.19E−04	5.16E−03
	External rotation	− 0.16	− 0.1944	1.39E−03			1		
*Patellofemoral Joint*									
Translations (mm)	Lateral shift	4.78	− 0.0433	3.71E−04	4.76	− 0.0252	0.121	2.33E−04	3.21E−03
	Anterior translation	49.59	− 0.0940	− 6.86E−04	49.63	− 0.0694	0.108	− 8.57E−04	− 2.09E−03
	Superior translation	13.21	− 0.2192	7.15E−04	13.26	− 0.1907	0.124	5.17E−04	− 2.54E−03
Rotations (deg)	Flexion	0.30	0.6284	6.49E−04	0.42	0.7115	0.382	6.71E−05	− 5.14E−03
	Lateral rotation	− 3.87	0.0019	2.34E−04	− 3.83	− 0.0558	− 0.371	6.71E−04	− 8.38E−03
	Lateral tilt	1.08	0.0328	− 2.90E−04	1.15	0.0851	0.244	− 6.58E−04	− 2.89E−03

3D tibiofemoral and patellofemoral kinematic data for all 6 activities and all 10 participants were pooled, and each model was then created by fitting second-order polynomial equations to these data

### Model Cross-Validation

**Figure 3:**
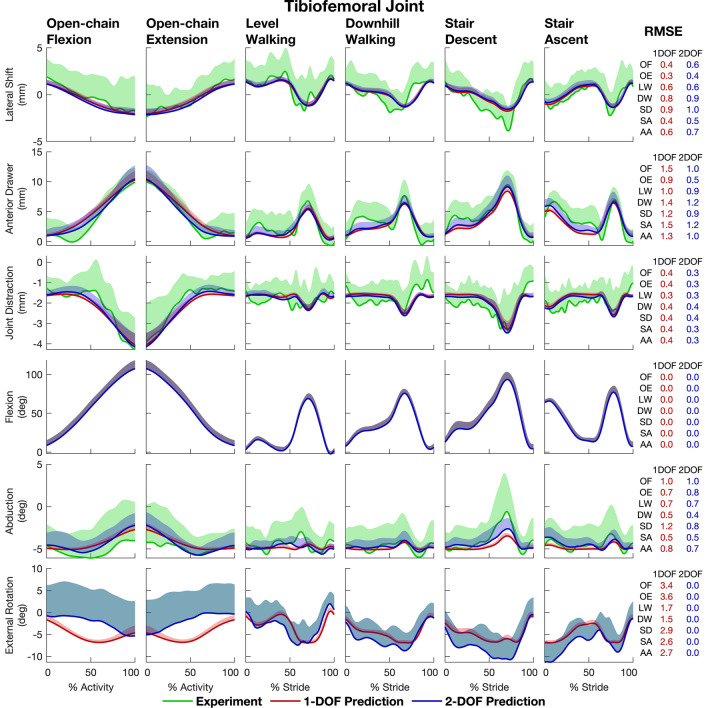
‘Leave-one-out’ model cross-validation results illustrating the accuracies of the 1-DOF and 2-DOF models in predicting tibiofemoral kinematics. The 1-DOF and 2-DOF models were fitted using data from all 6 activities and 9 participants pooled (54 trials), ‘leaving out’ the data from 1 participant. The fitted models were then used to predict the output kinematic parameters for each activity for the left-out participant. This procedure was performed 10 times, leaving out 1 participant each time, resulting in predictions of the kinematic parameters for all 10 participants. The mean (red and blue solid lines) and 1 standard deviation (shaded areas) of the predicted kinematic parameters for all 10 participants were then computed. The mean (green solid lines) and 1 standard deviation (shaded areas) of corresponding experimental results obtained from biplane X-ray imaging for the 10 participants are shown for comparison. Root-mean-square errors (RMSE) between the means of the predicted and measured kinematic parameters are given in the last column for each activity and for all activities pooled (labeled “AA”). *AA* all activities, *OF* open-chain flexion, *OE* open-chain extension, *LW* level walking, *DW* downhill walking, *SD* stair descent, *SA* stair ascent.

**Figure 4:**
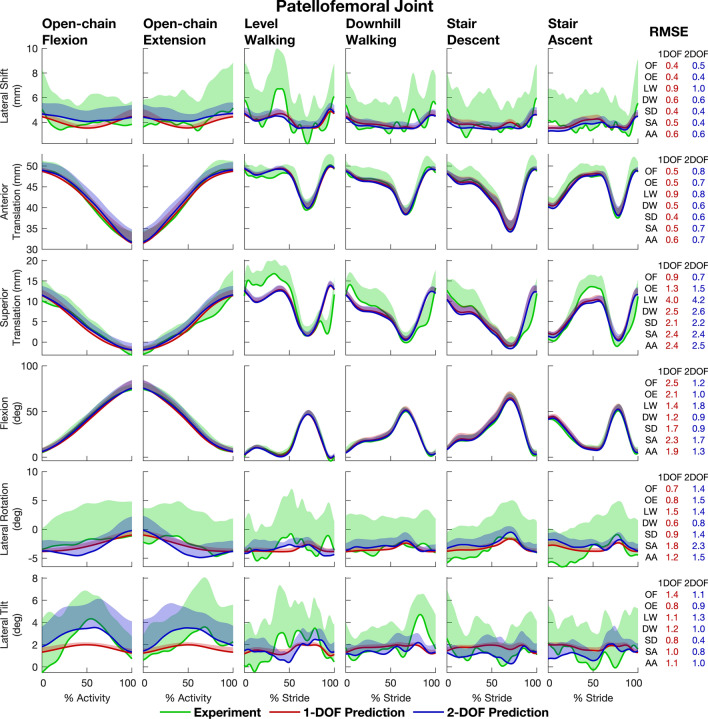
‘Leave-one-out’ model cross-validation results illustrating the accuracies of the 1-DOF and 2-DOF models in predicting patellofemoral kinematics. The 1-DOF and 2-DOF models were fitted using data from all 6 activities and 9 participants pooled (54 trials), ‘leaving out’ data from 1 participant. The fitted models were then used to predict the output kinematic parameters for each activity for the left-out participant. This procedure was performed 10 times, leaving out 1 participant each time, resulting in predictions of the kinematic parameters for all 10 participants. The mean (red and blue solid lines) and 1 standard deviation (shaded areas) of the predicted kinematic parameters for all 10 participants were then computed. The mean (green solid lines) and 1 standard deviation (shaded areas) of corresponding experimental results obtained from biplane X-ray imaging for the 10 participants are shown for comparison. Root-mean-square errors (RMSE) between the means of the predicted and measured kinematic parameters are given in the last column for each activity and for all activities pooled (labeled “AA”). *AA* all activities, *OF* open-chain flexion, *OE* open-chain extension, *LW* level walking, *DW* downhill walking, *SD* stair descent, *SA* stair ascent.

The 2-DOF model predicted 3D TF and PF kinematics more accurately than the 1-DOF model (Table 2, Figs. 3 and 4). At the TF joint, the mean RMS error for the 1-DOF model across all activities and all participants was 3.4°|mm compared to 2.4°|mm for the 2-DOF model (Table 2, Panel A). External rotation was associated with the largest RMS error for each participant when a 1-DOF model was used to predict 3D knee motion (range: 3.0°|mm to 10.0°|mm; mean: 5.8°|mm). Mean RMS errors for the 4 remaining TF kinematic parameters (i.e., abduction plus all three translations) were similar for the 1-DOF and 2-DOF models.

**Table 2:** Root-mean-square errors (RMSE) used to quantify the accuracy of model-predicted kinematics obtained from the ‘leave-one-out’ cross-validation analysis.

*Panel A: Tibiofemoral Joint*												
Participant left out from model fitting		P1	P2	P3	P4	P5	P6	P7	P8	P9	P10	Mean
*1-DOF Model (Input variable: tibiofemoral flexion)*												
Translations (mm)	Lateral shift	1.2	2.3	1.5	1.6	2.6	1.4	2.0	1.4	5.5	2.1	2.2
	Anterior drawer	3.0	5.1	1.5	2.7	2.3	2.2	2.0	1.7	2.5	2.8	2.6
	Joint distraction	1.6	1.0	0.7	0.9	1.0	1.0	1.2	1.0	0.9	1.0	1.0
Rotations (deg)	Flexion	0.0	0.0	0.0	0.0	0.0	0.0	0.0	0.0	0.0	0.0	0.0
	Abduction	3.6	2.3	1.1	2.2	2.6	3.4	4.6	2.9	3.6	3.3	3.0
	External rotation	10.0	7.3	6.2	5.8	5.4	5.9	3.0	5.0	5.6	3.8	5.8
All (deg|mm)		5.0	4.3	3.0	3.1	3.1	3.3	2.8	2.8	4.0	2.8	3.4
*2-DOF Model (Input variables: tibiofemoral flexion and external rotation)*												
Translations (mm)	Lateral shift	1.2	2.2	1.4	1.9	2.9	1.6	2.0	1.5	5.5	2.2	2.2
	Anterior drawer	2.5	4.5	2.1	2.5	2.9	2.0	1.6	1.5	2.9	2.4	2.5
	Joint distraction	1.5	1.1	0.7	1.0	1.0	1.0	1.3	0.9	1.0	1.0	1.1
Rotations (deg)	Flexion	0.0	0.0	0.0	0.0	0.0	0.0	0.0	0.0	0.0	0.0	0.0
	Abduction	4.1	1.5	1.6	1.4	3.1	3.4	4.4	2.6	4.3	3.0	2.9
	External rotation	0.0	0.0	0.0	0.0	0.0	0.0	0.0	0.0	0.0	0.0	0.0
All (deg|mm)		2.6	2.7	1.6	1.8	2.6	2.2	2.6	1.7	3.8	2.3	2.4

*Panel B: Patellofemoral Joint*												
Participant left out from model fitting		P1	P2	P3	P4	P5	P6	P7	P8	P9	P10	Mean
*1-DOF Model (Input variable: tibiofemoral flexion)*												
Translations (mm)	Lateral shift	2.9	1.9	2.3	1.4	5.2	1.5	2.3	1.3	3.5	1.6	2.4
	Anterior translation	0.9	1.9	0.9	2.4	4.1	1.5	4.0	1.2	3.4	2.5	2.3
	Superior translation	3.3	5.8	3.8	3.7	3.4	4.0	2.2	3.1	6.7	3.9	4.0
Rotations (deg)	Flexion	4.1	8.7	2.4	2.8	4.0	5.1	2.9	4.4	4.2	4.0	4.3
	Lateral rotation	12.2	6.5	2.7	8.5	4.0	4.1	2.7	6.7	4.2	3.2	5.5
	Lateral tilt	4.2	3.3	4.0	2.9	3.3	3.8	2.0	2.7	3.1	2.9	3.2
All (deg|mm)		5.8	5.3	2.9	4.3	4.1	3.6	2.8	3.8	4.3	3.1	4.0
*2-DOF Model (Input variables: tibiofemoral flexion and external rotation)*												
Translations (mm)	Lateral shift	2.5	1.7	2.0	1.4	5.2	1.5	2.4	1.5	3.9	1.7	2.4
	Anterior translation	2.0	1.3	1.5	2.0	3.9	1.5	4.5	1.6	3.1	2.4	2.4
	Superior translation	3.2	5.5	5.0	3.3	3.2	3.9	2.2	3.3	6.4	4.3	4.0
Rotations (deg)	Flexion	2.7	7.0	2.7	4.9	2.9	4.2	3.1	4.1	3.0	3.1	3.8
	Lateral rotation	12.0	6.0	4.3	7.5	4.9	4.9	2.8	6.8	5.6	3.7	5.9
	Lateral tilt	4.3	2.3	2.9	2.2	2.6	3.6	1.7	2.8	2.6	3.2	2.8
All (deg|mm)		5.6	4.6	3.3	4.1	3.9	3.5	2.9	3.8	4.3	3.2	3.9

Each RMSE represents the difference between a model-predicted kinematic parameter and the corresponding measurement obtained from biplane X-ray imaging. Each column with headings ‘P1’, ‘P2’, …‘P10’ gives the RMSEs for each of the 10 participants across all 6 activities. For example, for participant 1 (P1) the model was created using the kinematic parameters measured for all participants, except P1. The TF flexion angle measured for P1 was then used as an input to the model to predict all other kinematic parameters for P1. The last row in each panel (All) specifies the RMSE calculated by pooling the errors from all predicted kinematic parameters and all 6 activities for each participant. The last column (Mean) specifies the mean of the RMSE values calculated across all 10 participants

At the PF joint, mean RMS errors across all activities and all participants were 4.0°|mm and 3.9°|mm for the 1-DOF and 2-DOF models, respectively (Table 2, Panel B). Mean RMS errors for the 1-DOF and 2-DOF models were similar for each of the 6 PF kinematic parameters, with the largest error associated with lateral patellar rotation. Mean RMS errors for lateral patellar rotation were 5.5°|mm (range 2.7°|mm to 12.2°|mm) for the 1-DOF model and 5.9°|mm (range 2.8°|mm to 12.0°|mm) for the 2-DOF model.

RMS errors in the mean values of all kinematic parameters other than TF flexion and external tibial rotation were comparable for the 1-DOF and 2-DOF models for each of the 6 activities (compare the red and blue values in the last column of Figs. 3, 4). Differences in the RMS errors calculated for these 10 kinematic parameters ranged from 0.1 to 0.6°|mm (see rows labelled “AA” in the “RMSE” column in Figs. 3, 4).

## Discussion

The primary aim of this study was to determine the accuracy with which 1-DOF and 2-DOF kinematic models of the knee predict the 3D movements of the femur, tibia and patella for a wide range of daily activities. A multivariate regression analysis performed across 6 functional activities showed that a 2-DOF model with TF flexion and external tibial rotation as inputs describes the kinematic behavior of the knee-joint complex more accurately than a 1-DOF model with only TF flexion as the input (Fig. 2). Model cross-validation showed further that the 2-DOF model predicts 3D TF and PF joint kinematics more accurately than the 1-DOF model across all activities (Table 2, Figs. 3, 4), thus supporting our hypothesis.

The 1-DOF model was created by plotting TF and PF kinematic parameters against the TF flexion angle and using second-order polynomial equations to describe the relationships between each output kinematic parameter and the input TF flexion angle. A similar process was followed for the 2-DOF model, where each output kinematic parameter was plotted against two inputs: TF flexion angle and external tibial rotation. We selected these variables as input kinematic parameters for three reasons. First, the knee is often represented as a 1-DOF system where a single axis of rotation is used to reproduce the relative movements of the femur, tibia and patella.^3,11–14,16,17,22,24,26^ Second, TF flexion and external tibial rotation were used to define a 2-DOF model described previously by Hollister *et al*.^11^ and Churchill *et al*.^3^ Third, TF flexion and external tibial rotation represent the largest relative motions between the femur and tibia^23^ and are commonly measured using video motion capture and skin-mounted markers.^20^

There are many other combinations of TF kinematic parameters that could have been selected as inputs to a knee model. We used multivariate regression to investigate how well each of these different models describes the kinematic behavior of the knee-joint complex across all participants and all activities (Supplementary Material, Section S1). Specifically, second-order polynomial equations were used to fit the TF and PF kinematic data for 2-DOF, 3-DOF, 4-DOF and 5-DOF models with different combinations of TF kinematic parameters chosen as inputs. TF flexion was always included as one of these input kinematic parameters because it represents the primary motion of the knee joint. Thus, a 2-DOF model was represented by the TF flexion angle and one of the remaining five TF kinematic parameters (e.g., anterior tibial translation). Similarly, a 3-DOF model was represented by the TF flexion angle and two of the remaining five TF kinematic parameters (e.g., external tibial rotation and tibial abduction). This analysis yielded three important results. First, we found that from all possible combinations of input kinematic parameters used to define a 2-DOF model, the one with TF flexion and external tibial rotation as inputs gave the lowest residual (Fig. S1, top-left panel). Second, increasing the number of DOFs of the model beyond two brought diminishing returns as the magnitude of the residual decreased only slightly. Specifically, the residual was reduced by 0.9°|mm from 2.0 to 1.1°|mm when the number of DOFs was increased from one to two (Fig. S1, first column). However, increasing the number of DOFs from two to three reduced the residual by just 0.2°|mm, and increasing the number of DOFs further still to five reduced the residual by another 0.5°|mm. Third, increasing the number of DOFs of the model by using a larger number of TF kinematic parameters as inputs had a relatively small effect on the residuals calculated for the PF joint (Fig. S1, second column). The residual at the PF joint for a 1-DOF model with TF flexion as the input was 2.2°|mm compared to 1.7°|mm when the number of input parameters was increased to six (i.e., a total reduction of 0.5°|mm). By comparison, the residual at the TF joint decreased by 1.5°|mm when the number of input parameters was increased from one to five (Fig. S1).

Although we found that a 5-DOF model yields a lower residual than a 1-DOF model, this result is mainly of theoretical interest and has limited practical value. In practice, tibial abduction or any of the three TF translations are not likely to be used as inputs to a knee model because these kinematic parameters cannot be measured accurately using standard motion analysis techniques, such as video motion capture with skin markers. We suggest, therefore, that a 2-DOF model with two input parameters—the TF flexion angle measured about a mediolateral axis fixed in the femur (*X*_F_ in Fig. 1) and external tibial rotation measured about a longitudinal axis fixed in the tibia (*Z*_T_ in Fig. 1)—is the most suitable structure for describing the kinematic behavior of the knee-joint complex.

Each knee model was created by fitting second-order polynomials to 3D TF and PF kinematic data obtained from mobile biplane X-ray imaging. We also examined the effect of using polynomials of lower and higher order than two to fit the kinematic measurements (Supplementary Material, Section S2). The order of the polynomials had a relatively small effect on the residuals calculated at the TF and PF joints in each model (Fig. S2). For example, fitting the kinematic data using fourth-order rather than second-order polynomial functions reduced the residuals by less than 0.1°|mm at the TF joint and ~ 0.1°|mm at the PF joint. Thus, second-order polynomials were used in the current analysis as a compromise between the complexity of a model and its ability to accurately fit the 3D knee kinematic data.

One of the main findings of our study was that the 1-DOF model did not accurately predict external tibial rotation. The mean RMSE for external tibial rotation was 5.8°, whereas the errors associated with the four remaining kinematic parameters at the TF joint and all six kinematic parameters at the PF joint were similar for the 1-DOF and 2-DOF models (Table 2). We therefore recommend the 2-DOF for predicting 3D knee kinematics, even though the 1-DOF model with only one input kinematic parameter (TF flexion) may be more practical to implement. The reason is that measurements of internal–external tibial rotation obtained from standard gait analysis experiments are likely to be more accurate than the values of external tibial rotation predicted by the 1-DOF model described here. Using video motion capture with skin markers to measure 6-DOF knee kinematics across a wide range of activities, Richard *et al*.^20^ reported a mean RMS error for internal–external rotation of 2.7° (range 2.1°–3.8°), which is lower than the error in external tibial rotation predicted by the 1-DOF model.

**Figure 5:**
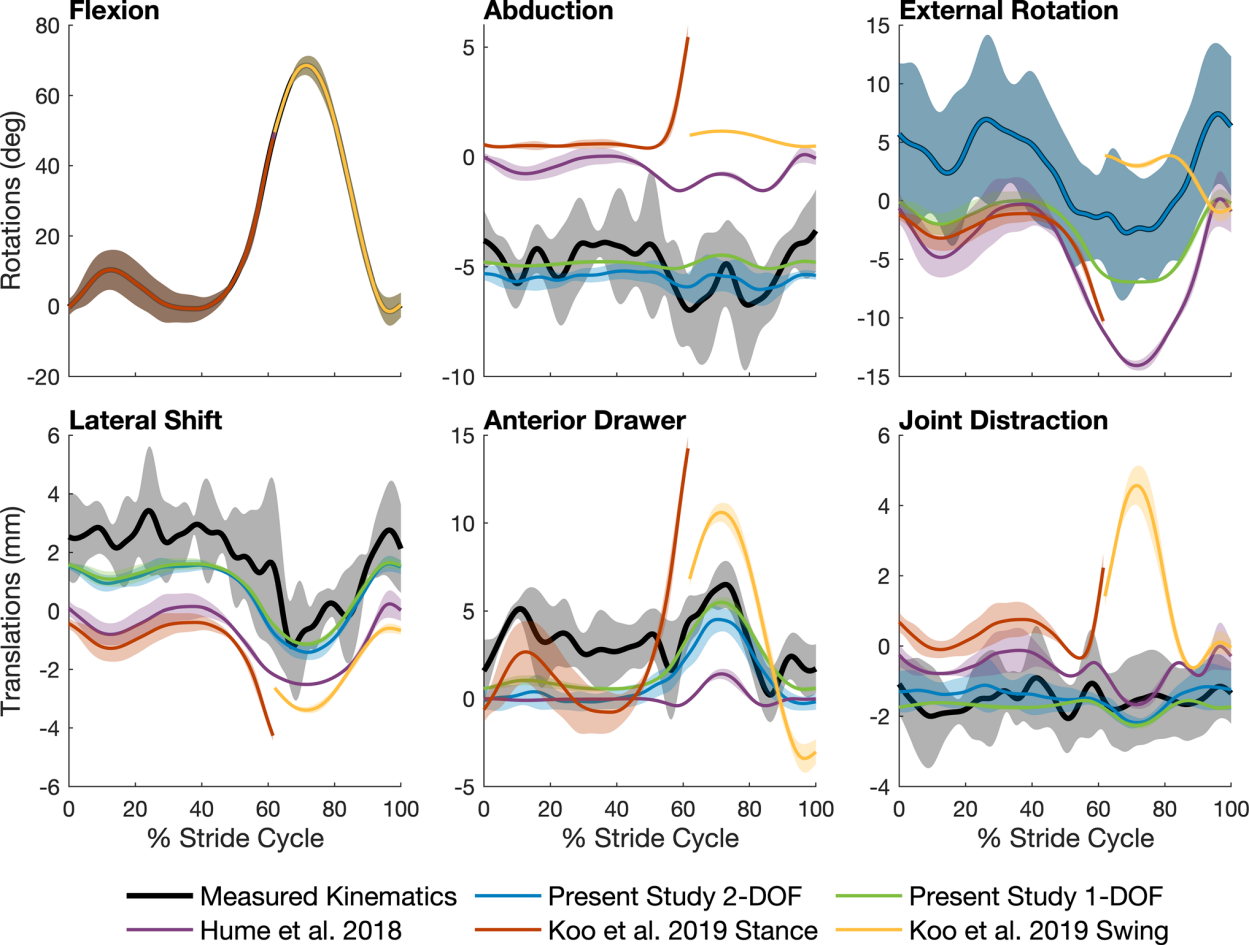
Comparison of tibiofemoral (TF) joint kinematic parameters predicted by models reported in the literature and those developed in the present study. The polynomial equations defining the 1-DOF and 2-DOF TF joint models developed in the present study are given in Table 1. The models drawn from the literature were the 1-DOF model developed by Hume *et al*.^12^ (see Table 2 in their paper); and the 1-DOF model developed by Koo and Koo^13^ (see Figs. 3 and 4 in their paper). TF kinematics for level walking measured for 5 participants (a subset of the cohort used in the study published by Gray *et al*.^9^) (black solid lines and gray shaded areas) were used to evaluate the accuracy of each model. Note that these 5 participants were not part of the cohort used in the present study. Each model was used to predict all output kinematic parameters of the TF joint (5 parameters for a 1-DOF model and 4 parameters for a 2-DOF model) for each of the 5 participants. The mean (colored solid lines) and 1 standard deviation (colored shaded areas) were then calculated for all 5 participants.

Two previous studies created 1-DOF knee models by fitting polynomial functions to the kinematic parameters describing the relative motion of the femur and tibia obtained from biplane X-ray imaging.^12,13^ We compared the performance of each of these models to that of the 1-DOF and 2-DOF models described in the present study (Table 1). Each model was used to predict all remaining secondary motions at the TF joint for one cycle of level walking, and model accuracy was assessed by comparing the predictions against *in vivo* 3D knee kinematic data measured from biplane X-ray imaging.^9^ The secondary motions of the tibia relative to the femur predicted by our 1-DOF and 2-DOF models were consistently within 1 standard deviation of the mean experimental data whereas the results obtained from the models reported in the literature were noticeably different (Fig. 5). The 1-DOF models reported by Hume *et al*.^12^ and Koo and Koo^13^ yielded predictions where the tibia was more abducted and medially shifted throughout the gait cycle as well as more internally rotated during swing. In addition, there were relatively small differences between the trajectories predicted by our 1-DOF and 2-DOF models. Although external tibial rotation predicted by the 1-DOF model remained within 1 standard deviation of the mean experimental data, the error in this kinematic parameter was ~ 5° over the entire gait cycle. This finding is consistent with the results obtained from the model cross-validation analysis and reinforces the fact that the main difference between the 1-DOF and 2-DOF models is the inability of the 1-DOF model to accurately predict external tibial rotation.

We found that all 6 PF kinematic parameters could be predicted to an accuracy of 4.0°|mm when TF flexion was the only input kinematic parameter used, and that a relatively small improvement (0.1°|mm) was obtained when a 2-DOF model was created with external tibial rotation added as an input (Table 2, Panel B). Adding external tibial rotation as an input improved the accuracies with which patellar flexion and lateral patellar tilt were predicted, but it also increased the errors in the predictions of anterior patellar translation and lateral patellar rotation. We conclude that patellar motion is more heavily influenced by the TF flexion angle than external tibial rotation. TF flexion predicted lateral patellar tilt, lateral patellar shift, and anterior patellar translation more accurately than patellar flexion, lateral patellar rotation, and superior patellar translation, which may be explained by the geometry of the PF joint. At TF flexion angles greater than ~ 20° the patella enters the femoral trochlea,^15^ and lateral patellar shift and tilt are then determined primarily by the shapes of the articulating surfaces of the patellar facets and trochlear groove. In contrast, patellar flexion, lateral patellar rotation, and superior patellar translation are influenced not only by the geometry of the PF joint but also by the activation of the quadriceps muscles and their lines of action.

It is important to note that the polynomial equations describing our 1-DOF and 2-DOF models (Table 1) must be used in conjunction with the joint coordinate system given in Fig. 1 because the kinematic parameters for the TF and PF joints in each model were defined in this coordinate system. For the same knee motion, values of kinematic parameters are likely to be different when a different joint coordinate system is used to define these parameters. To apply the equations in Table 1 to a knee model defined by a different joint coordinate system, a set of transformation matrices must be derived to relate the new coordinate system to the one in Fig. 1 to ensure that the correct knee motion is predicted. In Section S3 of the Supplementary Material, we derive the transformation matrices that relate our joint coordinate system to the one used in OpenSim.^4^ These transformation matrices allow the joint coordinate system specified in Fig. 1 to be applied in OpenSim. The equations given in Table 1 then may be used to calculate 3D TF and PF kinematics.

One limitation of the present study is that the 1-DOF and 2-DOF models were developed from knee kinematic data recorded for 6 activities of daily living performed by healthy young people. Caution is advised when using these models to predict knee kinematics for other activities (e.g., running) or another cohort of participants (e.g., healthy older people or individuals with conditions such as knee osteoarthritis). Another limitation of our results is that the RMS errors associated with the model-predicted knee kinematics are much larger than the accuracy with which the relative movements of the bones can be measured using biplane X-ray imaging. For example, the mean RMS error in predicting external tibial rotation using the 1-DOF knee model was 5.8° compared to an RMS error of 0.6° obtained from biplane X-ray imaging.^10^ The relatively high errors in the model predictions can be explained firstly by the observation that no two kinematic parameters at either the TF or PF joint are perfectly coupled to each other^23^; and secondly by the relatively high inter-participant variability in the knee kinematic data.^7^ In addition, the model predictions are inextricably linked to the reference frame assigned to each bone. Because the positions and orientations of the axes defining the reference frame are usually determined by bony prominences, errors in model-predicted knee kinematics ultimately depend on the accuracy with which reference frames can be assigned to an individual participant based on their anatomy. Finally, even though our 1-DOF and 2-DOF models were developed using participant-specific knee kinematic data, the only anatomical feature considered was the femoral bicondylar width. Using other anatomical features such as tibial slope, femoral condyle diameter, and patellar facet width to personalize the model may yield more accurate predictions of TF and PF joint motion for individual participants, but this approach is also likely to make implementation of the model more difficult.

In summary, we found that a 2-DOF model with TF flexion and external tibial rotation as inputs predicted 3D knee kinematics more accurately than a 1-DOF model with only TF flexion specified as an input. At the TF joint, mean RMS errors across all activities and all participants were 3.4°|mm and 2.4°|mm for the 1-DOF and 2-DOF models, respectively. At the PF joint, mean RMS errors across all activities and all participants were 4.0°|mm and 3.9°|mm for 1-DOF and 2-DOF models. These results indicate that a 2-DOF knee model with two rotations as inputs may be used with confidence to predict the full 3D motion of the knee-joint complex.

## Supplementary Information

Below is the link to the electronic supplementary material.

Supplementary file1 (PDF 513 kb)
