# Supplementary material for: A Two-Degree-of-Freedom Knee Model Predicts Full Three-Dimensional Tibiofemoral and Patellofemoral Joint Motion During Functional Activity
Source: Ann Biomed Eng. 2022 Sep 9;51(3):493–505. doi: 10.1007/s10439-022-03048-2 (PMC9928808; doi:10.1007/s10439-022-03048-2)
Supplement: Supplementary file 1 — Supplementary file1 (PDF 513 kb) [file 10439_2022_3048_MOESM1_ESM.pdf]

Supplementary Material

A TWO-DEGREE-OF-FREEDOM KNEE MODEL PREDICTS FULL THREE-DIMENSIONAL TIBIOFEMORAL AND PATELLOFEMORAL JOINT MOTION DURING FUNCTIONAL ACTIVITY

Shanyuanye Guan, Hans A. Gray, Lucas T. Thomeer, Marcus G. Pandy

Department of Mechanical Engineering, University of Melbourne, Parkville, Victoria 3010, Australia

S1. Effect of using different combinations of tibiofemoral kinematic parameters as input variables

This study examined how well two different models described the kinematic behavior of the knee-joint complex: a 1-degree-of-freedom (1-DOF) model with the tibiofemoral (TF) flexion angle as the input variable, and a 2-DOF model with the TF flexion and external tibial rotation angles as input variables. Because 6 kinematic parameters are needed to describe the relative movements of the femur and tibia, there are many other combinations of TF kinematic parameters that could also have been selected as input variables. In this section, we investigate how well various other combinations of TF kinematic parameters describe the kinematic behavior of the knee-joint complex. Because flexion-extension represents the primary motion of the knee, the TF flexion angle was always included as an input variable. Therefore, among all possible combinations of the 6 TF kinematic parameters, a total of 32 combinations that included the TF flexion angle were selected for this analysis.

Each combination of input variables defined a specific model, and for each of these models a second-order polynomial equation of the following form was fitted to the relevant kinematic parameters:

$$y = c_0 + \sum_{j=1}^{n} \left(c_{1j} x_j + c_{2j} x_j^2 \right) \tag{S1}$$

where x_j is a TF kinematic parameter used as an input variable to the model, n is the number of input variables to the model, and c_0 and c_{ij} are the coefficients of the polynomial. Participant-specific models were created by fitting 1,206 observations pooled from all 6 activities for each participant (201 time points per trial and 1 trial for each of the 6 activities). The coefficients of the polynomials were found using a least-squares method. Specifically, for each participant, 32 models were created using the 32 combinations of TF kinematic parameters, and root-mean-square residuals (RMSR) were calculated using equations (3) and (4) for the TF and PF joints, respectively, as given in the text. For each combination the mean and standard deviation of RMSRs were calculated across all 10 participants for the TF and PF joints (Fig. S1).

As seen in the top-left panel of Fig. S1 below, amongst all possible combinations of input kinematic parameters used to define a 2-DOF model, the one with TF flexion and external tibial rotation as inputs gave the lowest residual. Further, increasing the number of DOFs of the model beyond two did not have a significant effect on the residuals calculated at either the TF or PF joint (first and second columns in Fig. S1).

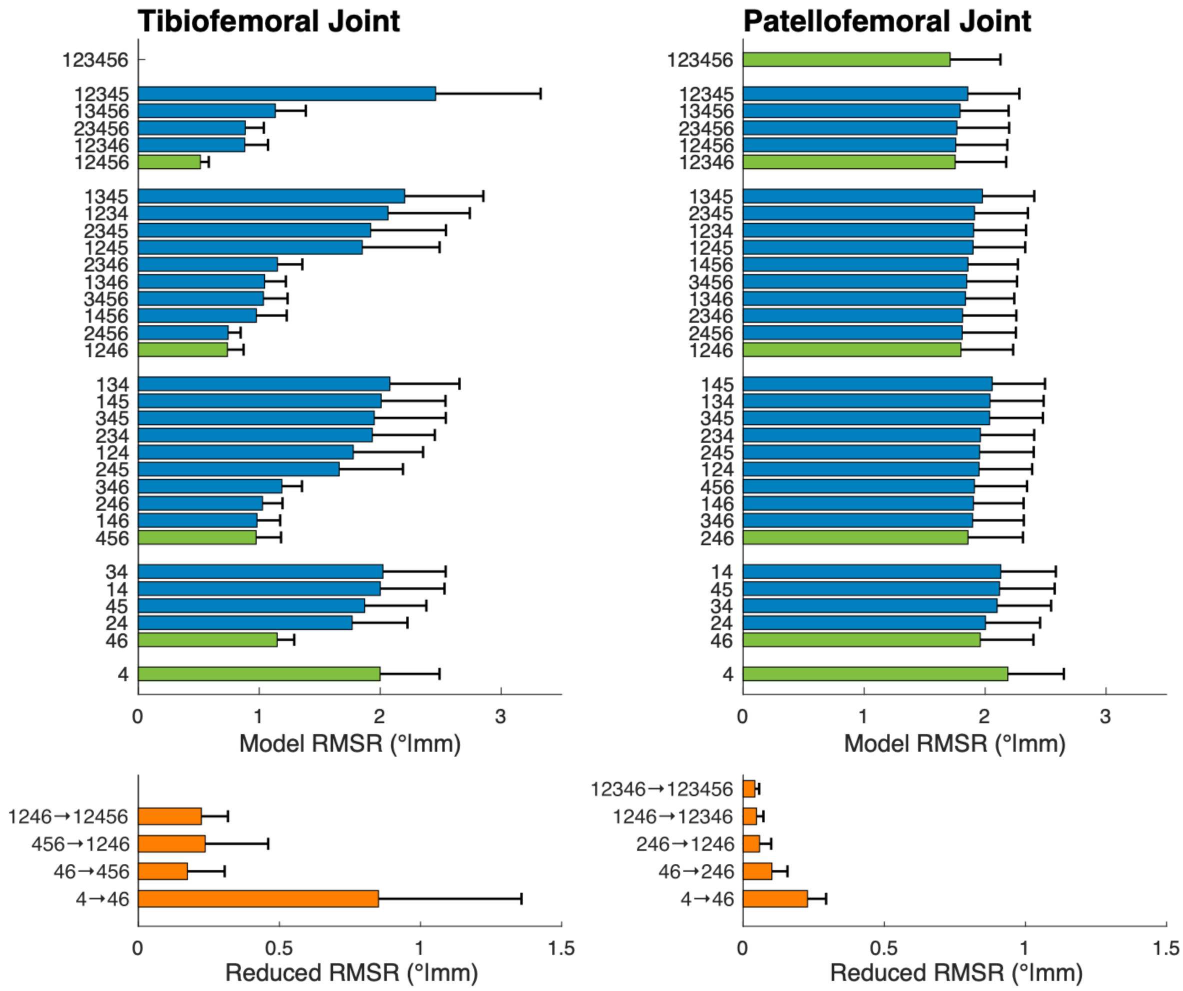

Figure S1. Root-mean-square residuals (RMSR) of models fitted for individual participants using tibiofemoral (TF) joint kinematics (left column) and patellofemoral (PF) joint kinematics (right column). For each participant, models using 32 combinations of TF kinematic parameters as input variables were fitted using data from all 6 activities pooled. Panels in the first row shows the mean (length of the colored bar) and standard deviation (error bar) of RMSRs calculated across all 10 participants. Models were grouped by the number of input variables for each model (i.e., the number of degrees-of-freedom of each model). The combination that resulted in the lowest RMSRs in each group are highlighted and shown as green bars in the first row. The TF kinematic parameters used to create each model were labelled as follows: 1 – lateral shift, 2 – anterior drawer, 3 – joint distraction, 4 – flexion, 5 – abduction, and 6 – external rotation. For example, '4' describes a 1-degree-of-freedom (1-DOF) model with only the TF flexion angle (parameter 4) used as the input variable. Similarly, '46' describes a 2-DOF model with the TF flexion and external tibial rotation angles used as the input variables. The panels in the second row shows the mean (length of the orange bar) and standard deviation (error bar) of the reduced RMSRs when the number of DOF of the model was increased by 1. For example, increasing the number of DOFs of the model from 1 to 2 reduced the RMSRs for the TF joint by 0.9°|mm (i.e., 0.9° or 0.9 mm) (left column) and also reduced the RMSRs for the PF joint by 0.2°|mm. Rotations of 1° and translations of 1 mm were equally weighted when calculating the RMSRs.

S2. Effect of using different orders of polynomial equations

First-order up to fourth-order polynomial equations were fitted to data for the tibiofemoral (TF) and patellofemoral (PF) joints to evaluate the effect of the order of the polynomial equation on fitting knee kinematics. For each fitted kinematic parameter (y), polynomial equations of the following forms were used for 1-DOF and 2-DOF models (equations (S2) and (S3), respectively):

$$y = c_0 + \sum_{i=1}^{m} \mathrm{c}_{i1} f^i \tag{S2}$$

$$y = \mathrm{c}_0 + \sum_{i=1}^{m} \left(\mathrm{c}_{i1} f^i + \mathrm{c}_{i2} e^i\right) \tag{S3}$$

where f is the TF flexion angle, e is the external tibial rotation, m is the order of the polynomial equation ($m = 1,2,3,4$), and c_0 and c_{ij} are the coefficients of the polynomial. Participant-specific 1-DOF and 2-DOF models were created by fitting 1,206 observations obtained by pooling the data from all 6 activities for each participant (201 time points per trial and 1 trial for each of the 6 activities). For each model, the coefficients of the polynomials were found using a least-squares method and RMSRs were calculated using equations (3) and (4) for the TF and PF joints, respectively, as given in the text. The mean and standard deviation of the RMSR were calculated across all 10 participants for the 1-DOF and 2-DOF models at the TF and PF joints (Fig. S2).

As illustrated in Fig. S2 below, the order of the polynomials had a relatively small effect on the residuals calculated at the TF and PF joints in each model. For example, fitting the kinematic data using fourth-order rather than second-order polynomial functions reduced the residuals by less than 0.1°|mm at the TF joint and ~0.1°|mm at the PF joint.

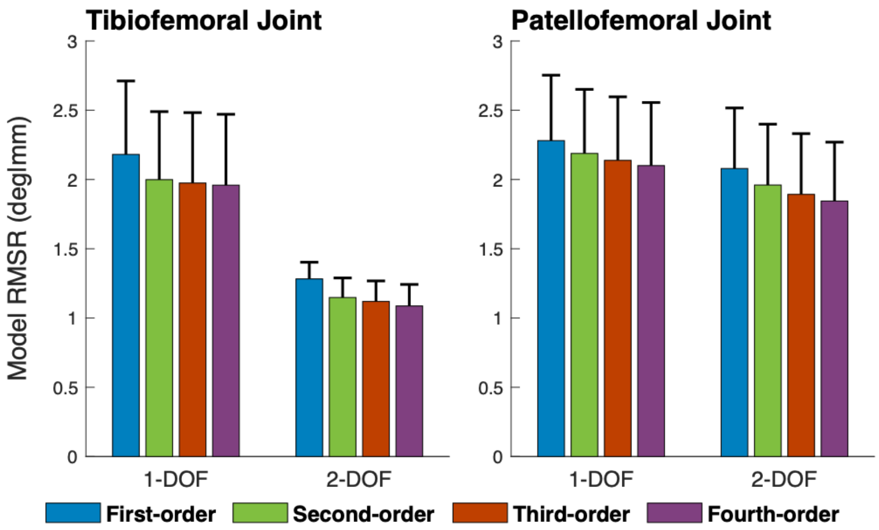

Figure S2. Root-mean-square residuals (RMSR) for 1-DOF and 2-DOF models fitted using first-order, second-order, third-order and fourth-order polynomial equations at the tibiofemoral and patellofemoral joints. Mean (height of the colored bar) and standard deviation (error bar) were calculated across all 10 participants. The order of each polynomial equation is represented by a different colour as indicated in the legend. Rotations of 1° and translations of 1 mm were weighted equally.

S3. Transformation between different reference frames

Kinematic parameters were defined based on the reference frame/joint coordinate system (i.e., axes fixed on the femur, tibia, and patella) described in Fig. 1 given in the text. The reference frame was defined based on anatomical features identified on geometric models generated using the CT scan of each bone, and henceforth will be referred to as the 'CT bone reference frame'. The equations of the 1-DOF and 2-DOF models given in Table 1 were based on the kinematic parameters defined in this CT bone reference frame. For some applications (e.g., a knee model created in OpenSim), the reference frames used to define the orientation of the knee bones may be different from the ones used in the present study. Here, we develop the relevant transformation matrices to allow the reader to move between different reference frames and to use the polynomial equations given in Table 1 of the text. To illustrate a specific example, below we derive the transformation matrices relating the reference frame used in the present study to that adopted in OpenSim ("gait2392_simbody.osim").

The transformation matrices were derived using the positions of skin-mounted markers measured by the video motion capture system. For each trial collected from one participant in the present study (e.g., one trial of level walking), X-ray-based kinematic data were resampled to the same frequency as the motion capture data, so that at each time point during one gait cycle the motion capture data were paired with the corresponding biplane X-ray imaging data. For each time point, coordinates (p_{CT}) of markers on each bone (e.g., femur) in the CT reference frames were calculated using the following equation:

$$p_{\mathrm{CT}} = M_{\mathrm{X2CT}} M_{\mathrm{M2X}} p_{\mathrm{M}} \qquad \text{(S4)}$$

where p_{M} (in the format of $(x, y, z, 1)^{\mathrm{T}}$) is a vector containing the coordinates of a marker recorded by the motion capture system and expressed in the reference frame of the motion capture system; M_{M2X} is a 4×4 transformation matrix relating the motion capture system reference frame to the X-ray imaging system reference frame; and M_{X2CT} is a transformation matrix relating the X-ray imaging system reference frame to the CT bone reference frame (Fig. S3A). Coordinates (p_{OS}) of markers on each bone in the OpenSim reference frames were obtained from the OpenSim model (Fig. S3C). Prior to extracting coordinates, the OpenSim model of each participant was scaled based on a static standing trial. Given the coordinates of at least three markers expressed in both the OpenSim reference frame and CT bone reference frame, the transformation (M_{OS2CT}) between the OpenSim reference frames and the CT bone reference frames were calculated using the method described by Challis[1] and Söderkvist and Wedin[2] (Fig. S3B).

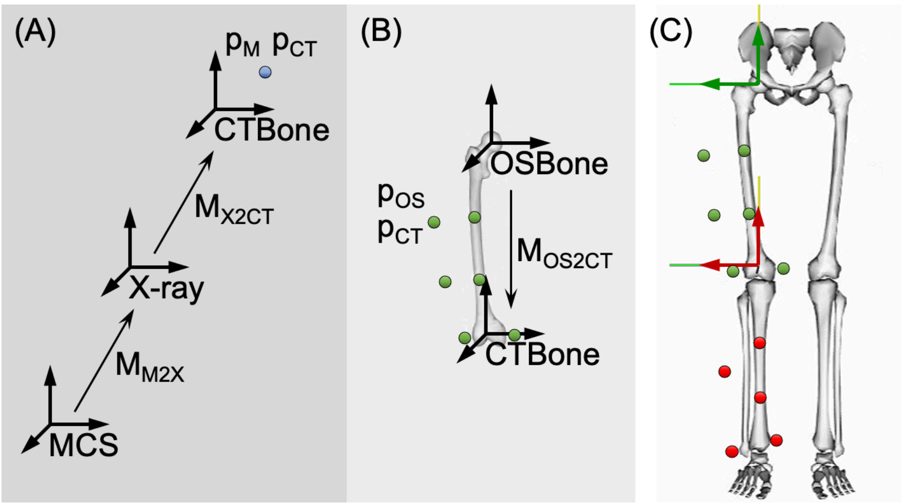

Figure S3. (A) Transformation between reference frames defined for the motion capture system (MCS), biplane X-ray system, and CT bone (CTBone). The blue dot represents a marker with coordinates P_{M} and P_{CT} in the MCS and CT bone reference frames, respectively. (B) Transformation between reference frames defined for the OpenSim bone (OSBone) and CT bone. Green dots represent markers attached to the skin of the right thigh. The coordinates of each marker are represented by P_{OS} and P_{CT} in the OpenSim bone and CT bone reference frames, respectively. (C) OpenSim bone reference frames for the right femur (green axes fixed on the femur) and right tibia (red axes fixed on the tibia). Green and red dots represent markers attached to the skin of the right thigh and the right shank, respectively. In the OpenSim model,

the coordinates of the thigh markers are given in the femoral reference frames (green axes), while those of the shank markers are given in the tibial reference frame (red axes).

The above method was used to calculate the average transformation matrices for the right femur ($M_{\mathrm{OS2CT\_Femur}}$) and right tibia ($M_{\mathrm{OS2CT\_Tibia}}$) of all 10 participants based on the data collected for their level walking trials:

$$M_{\mathrm{OS2CT\_Femur}} = \begin{bmatrix} -0.3401 & 0.1795 & 0.9231 & -5.2 \\ 0.9029 & -0.2122 & 0.3739 & 24.5 \\ 0.2630 & 0.9606 & -0.0899 & -0.2 \\ 0 & 0 & 0 & 1 \end{bmatrix} \quad \text{(S5)}$$

$$M_{\mathrm{OS2CT\_Tibia}} = \begin{bmatrix} 0.1608 & 0.0061 & 0.9870 & -9.6 \\ 0.9841 & 0.0761 & -0.1608 & 33.3 \\ -0.0761 & 0.9971 & 0.0062 & 8.7 \\ 0 & 0 & 0 & 1 \end{bmatrix} \quad \text{(S6)}$$

The transformation matrix $M_{\mathrm{OS2CT\_Tibia}}$ transforms coordinates in the OpenSim tibial reference frame (indicated by the red axes in Fig. S3C) to the CT-based tibial reference frame. The OpenSim femoral reference frame (indicated by the green axes in Fig. S3C) was located at the proximal end of the femur, and specifically at the center of the femoral head. By comparison, the CT-based femoral reference frame (Fig. 1) was located at the distal end of the femur. Hence, the transformation matrices relating the CT-based and OpenSim femoral reference frames also depend on the length of femur for each participant. To eliminate the effect of variations in femur length between each participant, we defined an OpenSim reference frame fixed on the distal femur coincident with the OpenSim tibial reference frame (red axes in Fig. S3C) when the right knee is fully extended (i.e., the value of “knee_angle_r” is zero) in the OpenSim model. Thus, the transformation matrix $M_{\mathrm{OS2CT\_Femur}}$ in equation (S5) transforms all coordinates defined in the OpenSim femoral reference frame located at the distal femur to the CT-based femoral reference frame.

References

1. Challis J. H. A procedure for determining rigid body transformation parameters. *Journal of biomechanics*. 28(6):733-737, 1995.
2. Söderkvist I. and P.-Å. Wedin. Determining the movements of the skeleton using well-configured markers. *Journal of biomechanics*. 26(12):1473-1477, 1993.
